# Study on the Performances of Waste Battery Powder Modified Asphalt and Asphalt Mixture

**DOI:** 10.3390/polym14245409

**Published:** 2022-12-10

**Authors:** Xinli Gan, Peng Chen, Bin Yu, Wengang Zhang

**Affiliations:** 1 School of Transportation Engineering, Guizhou Institute of Technology, Guiyang 550003, China; 2 Beijing CCCC Qiaoyu Science and Technology Co., Ltd., Beijing 271000, China; 3 School of Civil and Architectural Engineering, Shandong University of Technology, Zibo 255049, China

**Keywords:** WBP, modified asphalt, dynamic shear rheological properties, full section fracture energy, technical performance

## Abstract

As an asphalt modifier, waste battery powder (WBP) has been proven to be possible. This paper studies the modification effect of WBP on asphalt. The Flight Test Instrumentation Requirements (FITR) of WBP, Dynamic Shear Rheology (DSR) test, and Full Section Fracture Energy Test (FSFET) of asphalt are carried out. The high-temperature rheological properties and low-temperature properties of WBP modified asphalt are analyzed. The high-temperature stability, low-temperature crack resistance and water stability of WBP modified asphalt mixture are tested. The research results show that the modification of asphalt by WBP is essentially physical modification but the mixing of WBP has a certain enhancement effect on the bond energy of the methylene group, which is helpful to improve the technical performance of modified asphalt. The proportion of elastic components in asphalt can be significantly increased by adding WBP, thus enhancing the deformation resistance of asphalt under high-temperature conditions. The dynamic shear modulus of 10% waste battery powder is about 1.5–2.0 times that of 0% waste battery powder. The mixing of WBP reduces the proportion of viscous components in asphalt which is unfavorable to the crack resistance under low temperatures. The greater the amount of WBP, the smaller the fracture energy density, the content of WBP is 6% and 10%, the fracture energy density is about 60–80% and 40–60% of the original asphalt, and the low temperature cracking resistance of asphalt decreases. The modification effect of WBP on asphalt is much lower than that of SBS.

## 1. Introduction

China is the largest battery producer in the world, with an annual output of more than 20 billion, of which disposable batteries are the main type [1]. Waste batteries have become one of the fastest-growing forms of garbage with a growth rate of 20% [1]. 96% of the batteries produced in China are zinc manganese batteries and alkaline manganese batteries, whose main components are manganese, mercury, zinc, and other heavy metals [2,3]. Whether in the atmosphere or buried deep in the ground, the heavy metal components of waste batteries will overflow with the leachate, causing the pollution of groundwater and soil, and will seriously endanger human health over time. Most countries in the world have established relatively perfect recycling systems for waste batteries. For example, Germany has implemented a deposit system for purchasing mercury batteries. When consumers return old batteries, the deposit will be returned, and waste batteries will be sent to recycling plants for recycling. The United States has also implemented the recycling system of waste batteries and mercury-free primary batteries. Japan has not only established a sound waste battery recycling system but also realized the development and manufacturing of waste battery secondary products, forming a complete industrial chain. At present, in China, the vast majority of waste batteries are still treated as domestic waste and are treated in a unified way. The main treatment methods are landfilling and incineration. Such treatment methods have problems such as high pollution and low efficiency. Therefore, the recovery and secondary utilization of waste batteries in China is still in its infancy. In fact, waste batteries contain a variety of non-renewable disposable resources. For example, 3000 tons of waste batteries can recover 141 tons of impure zinc ingots, 300 tons of metallurgical manganese dioxide, 181 tons of electrolytic zinc, 340 tons of electrolytic manganese dioxide, and 500 tons of iron sheet, which is equivalent to the cost of developing two medium-sized mines [4,5,6].

In addition, many main components of waste batteries can improve the performance of asphalt, and WBP can be used as a modifier of asphalt. For example, Zinc Oxide (ZnO) in waste batteries has been reported in the relevant literature as improving the PG, fatigue life, and rutting resistance of asphalt binder [2]. The composite modifier composed of nano ZnO and SBS can significantly improve the high-temperature performance and shear resistance of asphalt, and the colloidal structure of asphalt is more stable [3]. Zinc oxide/layered silicate composite modified asphalt has good compatibility and asphalt storage stability, which can comprehensively improve the thermal oxidation resistance and ultraviolet ageing resistance of asphalt [7,8].

WBP contains manganese oxide (MNO). Zakieva et al. [9] reported that manganese ions, taking part in high-temperature oxidation-reduction reactions of modifier compounds containing acyl radicals and double bonds, are capable of forming oxy-radicals in the presence of air oxygen. This can effectively improve the bonding performance and low-temperature performance of asphalt. In addition, MNO can significantly improve the ability of asphalt to resist permanent deformation. Some scholars also used manganese dioxide as filler in asphalt mixture and found that manganese dioxide can improve the elasticity of asphalt mastic macadam, thus improving its high-temperature stability. At the same time, manganese dioxide can absorb microwave energy and generate heat, making asphalt mixture the ability to induce healing [10,11].

The WBP contains a large amount of graphite. Relevant research reports show that graphite can enhance the high-temperature delayed elastic creep recovery property, creep stiffness modulus [12,13], creep rate [14,15,16], and glass transition temperature of the asphalt [17,18,19,20,21]. The layered structure of graphene blocks the penetration of oxygen into asphalt, inhibits the volatilization of light components of asphalt, and enhances its anti-aging performance [22,23]. Graphene can also improve the performance of SBS modified asphalt. After adding graphene, the mechanical strength and deformation recovery ability of SBS modified asphalt is increased, and the rutting resistance is improved [24,25] with graphene and SBS modified asphalt forming a stable physical crosslinking state. In addition, graphene has no significant effect on the low-temperature performance of asphalt [26,27].

In addition to the above substances, there are Li, Fe, Cd, Ni, and other elements in the WBP [28,29]. There are few studies on the modification of asphalt by WBP composed of so many elements. It is unclear which performance of asphalt can be improved by WBP, which performance can be damaged to some extent, and what the level of technical performance of asphalt modified by WBP is [30,31].

So, in this paper, the FITR test of WBP, dynamic shear rheology test, and full section fracture energy test of asphalt are carried out. The high-temperature rheological properties and low-temperature properties of WBP modified asphalt are analyzed. The high-temperature stability, low-temperature crack resistance, and water stability of WBP modified asphalt mixture are tested. The difference between this paper and other related references is that the waste battery powder is used to modify asphalt, rather than the modification of a single component, which has better feasibility for large-scale utilization in the future. In addition, the scientific and technological innovation of this paper is to study the performance of waste battery powder in asphalt, and reveal the modification mechanism, which provides detailed data for later research on WBP modified asphalt mixture and has a good reference significance.

## 2. Materials and Methods

### 2.1. Raw Materials

#### 2.1.1. WBP

**Figure 1:**
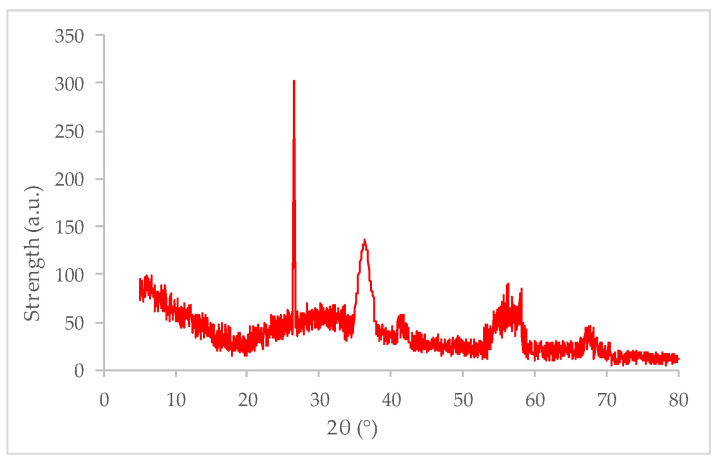
XRD spectrum of WBP.

The battery used in this paper is produced by Shandong Huatai New Energy Battery Co., Ltd (Linyi, China). After the external packaging of the waste battery was removed, take the positive material to dry, and use 0.075 mm sieve to sieve for standby. The main components of positive materials are carbon black and manganese-containing oxides, accounting for 48% and 51% of the total mass of powder, respectively. In addition, a small amount of Zn and other oxides are obtained. The prepared WBP was black powder. The X-ray diffraction (XRD) is produced by Suzhou Langsheng Scientific Instrument Co., Ltd (Xuancai, Suzhou, China) test was carried out under the conditions that the tube current is 40 mA, the tube voltage is 40 kV, and the diffraction angle 2θ is 5~80°, scanning frequency 8 (°) ∙ min^−1^. The XRD spectrum is shown in Figure 1.

According to the XRD spectrum, there is a strong diffraction peak when 2θ is 26.7° and there is no other impurity peak, indicating that one of the main components in the battery powder is carbon with high cleanliness. In addition, the diffraction peak also appeared when 2θ is 56.6°, which is the diffraction peak of various valence oxides of Mn, indicating that various valence oxides of Mn are also one of the main components of battery powder. The diffraction peaks (ZnO) appeared when 2θ is 36.2° or 67.9°, and the reason for the appearance is related to the invasion of cathode material to positive material.

#### 2.1.2. Asphalt

The 50^#^, 70^#,^ and 90^#^ A-grade road asphalt selected in this paper was produced by Sinopec Qilu Petrochemical Company (Zibo, China). SBS Ι-C polymer modified asphalt was prepared based on 90^#^ Grade A road-petroleum asphalt. SBS Ι-C polymer modified asphalt was used to compare the performance with WBP modified asphalt. Table 1 is the technical indicators of asphalt.

#### 2.1.3. Aggregate

The aggregate used in this paper is broken from basalt (Shandong Hongju Resources Recycling Co., Ltd, Zibo, China), and the mineral powder is ground from limestone (Shandong Hongju Resources Recycling Co., Ltd, Zibo, China),. The technical indicators are shown in Table 2, Table 3 and Table 4.

### 2.2. Preparation of WBP Modified Asphalt

**Figure 2:**
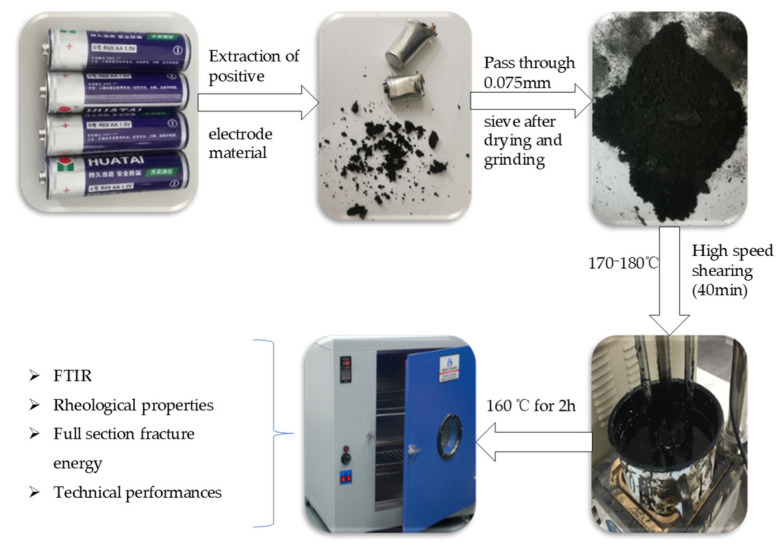
The preparation process of WBP modified asphalt (adding, shear and mixing, development).

The WBP is prepared for standby according to the method described in Section 2.1.1; then the asphalt is heated to 170–180 °C and the battery powder is slowly added into the asphalt. The high-speed shear apparatus is used to shear at 1000 r/min for 10 min at low speed, and then shear at 5000 r/min for 30 min at high speed. The asphalt with WBP is put into an oven at 160 °C to develop for 2 h, and the preparation of WBP modified asphalt is completed. The preparation process is shown in Figure 2.

### 2.3. Experimental Design

In this paper, 50^#^, 70^#,^ and 90^#^ A-grade road asphalt were used as raw materials, and 2%, 6%, and 10% WBP of asphalt mass were respectively mixed into asphalt to prepare WBP-modified asphalt by the method described in Section 2.2. The modified asphalt with WBP was tested by the Flight Test Instrumentation Requirements (FTIR) (FTIR650, Tianjin Gangdong SCI. & Tech. CO,. LTD, Tianjin, China) to acquire the infrared spectral image and analyze the characteristic peak, and the modified mechanism of WBP on asphalt was analyzed based on the test results. Dynamic shear rheological test (DSR) was carried out on the prepared WBP-modified asphalt, and the Dynamic shear modulus (G*), phase angle (δ), and rutting factor G*/sin(δ) were acquired; then the high-temperature rheological property was analyzed. The full section fracture energy test (FSFET) was also carried out on the prepared WBP modified asphalt, the calculated value of fracture energy density in whole section of asphalt with different content of WBP was obtained, and then the low-temperature crack resistance was analyzed. In addition, the conventional performance test of WBP modified asphalt mixture was also carried out. The dynamic stability (DS), maximum bending tensile strain (ε_B_), freeze-thaw splitting strength ratio (TSR) and residual stability (MS′) of the asphalt mixture were obtained. Then the pavement performances of WBP modified asphalt mixture were analysed [32,33,34].

## 3. Results

### 3.1. FTIR

**Figure 3:**
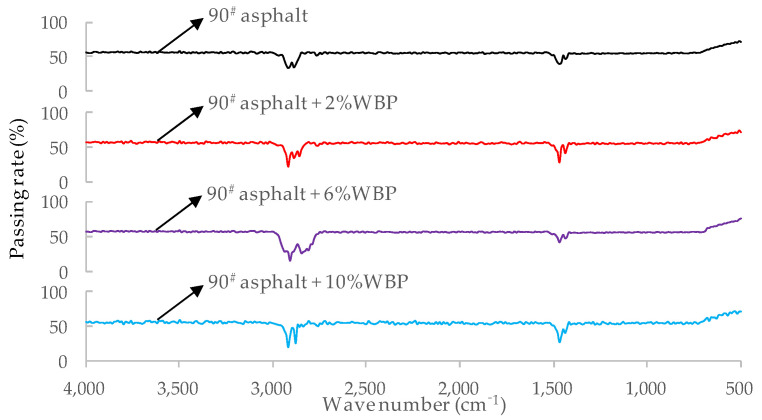
FTIR test results of 90^#^ asphalt with different content of WBP (90^#^ + 0%WBP, 90^#^ + 2%WBP, 90^#^ + 6%WBP, 90^#^ + 10%WBP).

In view of the similar mechanism (physical and chemical reaction) of the modification of WBP and various grades of asphalt, based on reducing the test amount, this paper uses FTIRS to test only 90^#^ asphalt and the modified asphalt mixed with WBP of 2%, 6%, and 10% into 90^#^ asphalt. The test results are shown in Figure 3.

Comparing the FTIR test results of 90^#^ asphalt and 90^#^ asphalt mixed with different content of WBP, it is not difficult to find that their characteristic absorption peaks are similar; that is, no new characteristic absorption peaks are generated, and the original characteristic peaks are not disappeared. This shows that the modification of WBP on asphalt is essentially a physical modification. It can be seen from Figure 3 that the characteristic peaks near 2930 cm^−1^ (strong peak) and 1470 cm^−1^ are obvious, and these two characteristic peaks correspond to methylene. With the increase of the content of WBP, the 2930 cm^−1^ absorption peak also increases, which indicates that the incorporation of WBP has a certain enhancement effect on the bond energy of the methylene group (==CH_2_). Bond Energy is a physical quantity that measures the strength of chemical bonds from the energy factor. For polyatomic molecules, the bond energy is the energy absorbed by the complete dissociation of 1 mol gaseous molecules into gaseous atoms and distributed to each covalent bond in the structural formula. The higher the bond energy, the lower the energy of the material itself. The smaller the bond energy, the higher the energy of the material itself. Because the energy is low, the structure of the material itself is stable, needing to absorb more heat, and the bond energy is large. The energy is high, the structure of the material itself is unstable, the heat to be absorbed is low, and the bond energy is small. The enhancement of the bond energy of methylene group will further enhance the molecular force between asphalt and WBP, which will help to improve the technical performance of modified asphalt.

### 3.2. High Temperature Rheological Property

**Figure 4:**
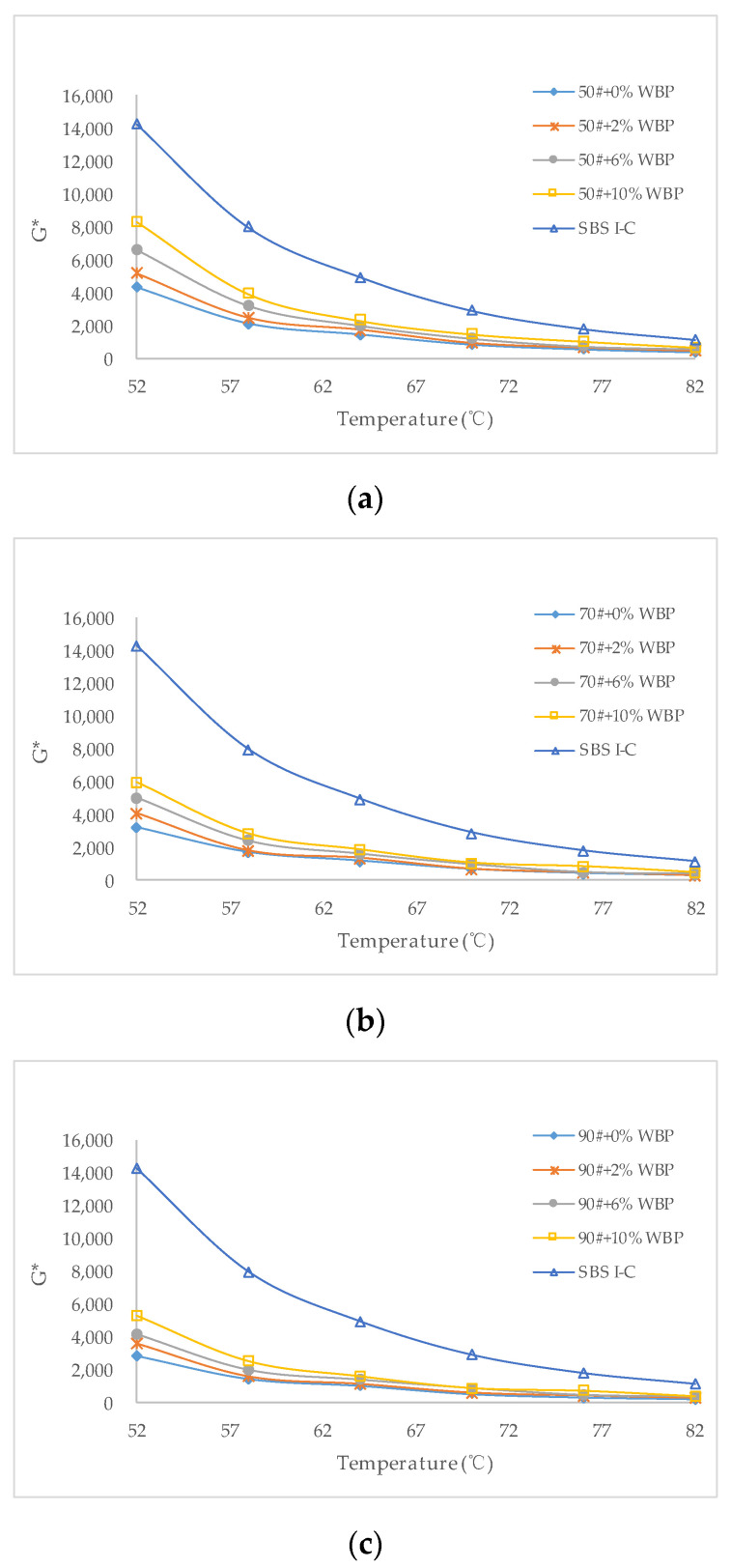
G* of asphalt with different WBP content: (**a**) 50^#^ + 0%WBP, 50^#^ + 2%WBP, 50^#^ + 6%WBP, 50^#^ + 10%WBP, SBS Ι-C; (**b**) 70^#^ + 0%WBP, 70^#^ + 2%WBP, 70^#^ + 6%WBP, 70^#^ + 10%WBP, SBS Ι-C; (**c**) 90^#^ + 0%WBP, 90^#^ + 2%WBP, 90^#^ + 6%WBP, 90^#^ + 10%WBP, SBS Ι-C.

In this paper, the dynamic shear rheometer was used to scan the 50^#^, 70^#^, and 90^#^ asphalt with different WBP content, and SBS modified asphalt was introduced as a comparison. The scanning temperature range was 52–82 °C. Dynamic shear modulus (G*), phase angle (δ), and rutting factor G*/sin(δ) were acquired. G* can be used to characterize the elastic components in asphalt. In general, the larger the G* value, the higher the proportion of elastic components in asphalt, and the stronger the asphalt’s ability to resist shear deformation at high temperatures. The phase angle δ indicates the proportion of viscous components in asphalt. The larger the viscous component, the worse the asphalt’s ability to resist shear deformation at high temperatures. G*/sin(δ) is generally used to evaluate the ability of asphalt to resist permanent deformation. The larger the value, the better the ability of asphalt to resist permanent deformation. G* of different asphalt with different WBP content is shown in Figure 4.

Figure 4 shows that the G* of various asphalts decreases rapidly with the increase in scanning temperature. For 50^#^ asphalt, the greater the content of WBP, the greater the G* at the same scanning temperature. The G* of asphalt with 10% WBP is about 1.5–2.0 times that of asphalt with 0% WBP. 70^#^ asphalt and 90^#^ asphalt also have the same rule, which shows that the proportion of elastic components in asphalt can be greatly increased by adding WBP. Combined with the results of FTIR, the absorption peak of asphalt is enhanced near 2930 cm^−1^ after the addition of WBP. It is not difficult to analyze that the aromatic C==C vibration is enhanced and the C==C double bond itself has higher bond energy; and its further enhancement is bound to enhance the anti-deformation ability of asphalt under high-temperature conditions.

Compared with 50^#^, 70^#^, and 90^#^ asphalt, when the content of WBP is the same, under the same scanning temperature, the G* of 50^#^ asphalt is the largest, followed by 70^#^ asphalt, and 90^#^ asphalt as the smallest. But by comparing SBS Ι-C modified asphalt, even if it is 50^#^ asphalt mixed with 10% WBP, it still cannot reach the level of SBS Ι-C modified asphalt. At the same scanning temperature, the G* of SBS Ι-C modified asphalt is much higher than that of 50^#^ asphalt with 10% WBP. This also suggests that WBP has a certain effect on improving the high-temperature performance of asphalt, but it is far from achieving the modified effect of SBS.

**Figure 5:**
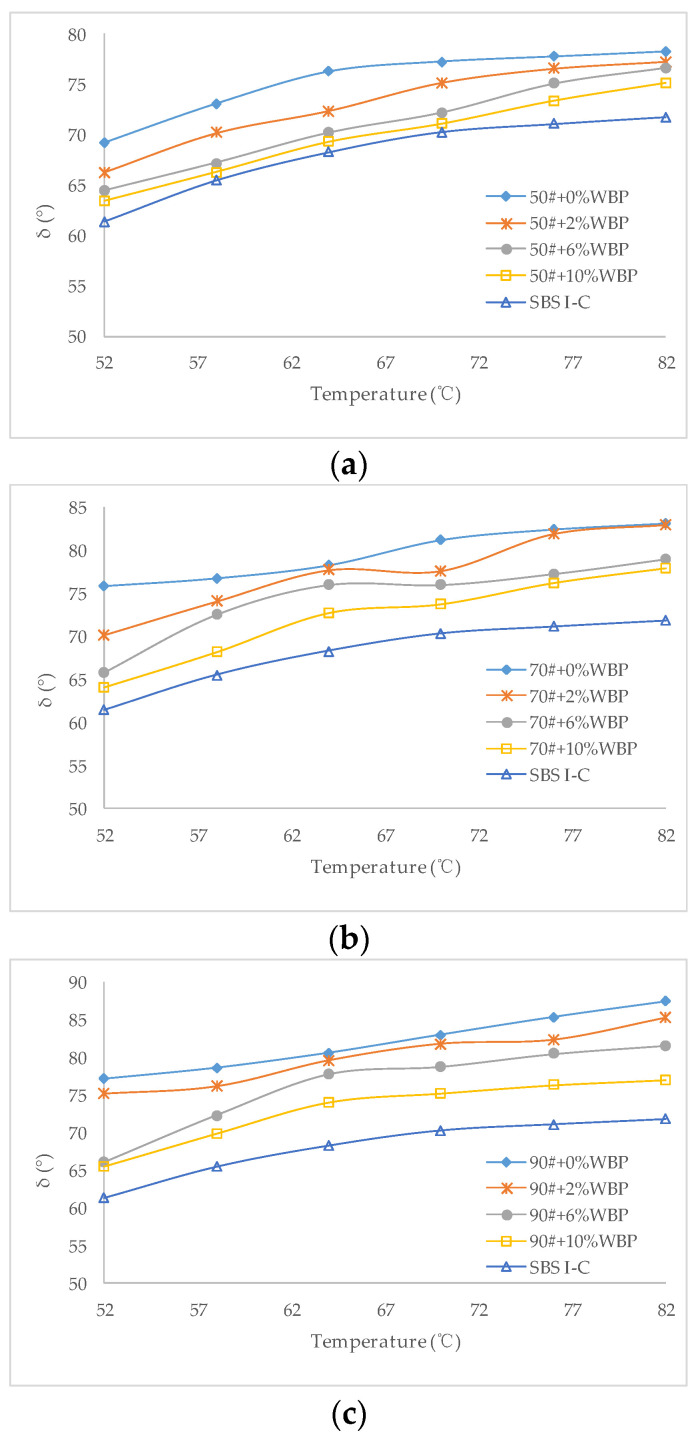
δ of asphalt with different WBP content: (**a**) 50^#^ + 0%WBP, 50^#^ + 2%WBP, 50^#^ + 6%WBP, 50^#^ + 10%WBP, SBS Ι-C; (**b**) 70^#^ + 0%WBP, 70^#^ + 2%WBP, 70^#^ + 6%WBP, 70^#^ + 10%WBP, SBS Ι-C; (**c**) 90^#^ + 0%WBP, 90^#^ + 2%WBP, 90^#^ + 6%WBP, 90^#^ + 10%WBP, SBS Ι-C.

The δ of different asphalt with different WBP content is shown in Figure 5.

Figure 5 shows that the δ of various asphalts increases gradually with the increase of scanning temperature. For 90^#^ asphalt, the greater the content of WBP, the smaller the δ at the same scanning temperature. 70^#^ asphalt and 50^#^ asphalt also have the same rule. This shows that the mixing of WBP reduces the proportion of viscous components in asphalt, which is beneficial to the ability of asphalt to resist permanent deformation at high temperatures, but unfavorable to crack resistance at low temperatures. Compared with 50^#^, 70^#^ and 90^#^ asphalt, when the content of WBP is the same, at the same scanning temperature, the δ of 50^#^ asphalt is the smallest, followed by 70^#^ asphalt, with 90^#^ asphalt being the largest. But it is much higher than the δ of SBS Ι-C modified asphalt at the same scanning temperature.

**Figure 6:**
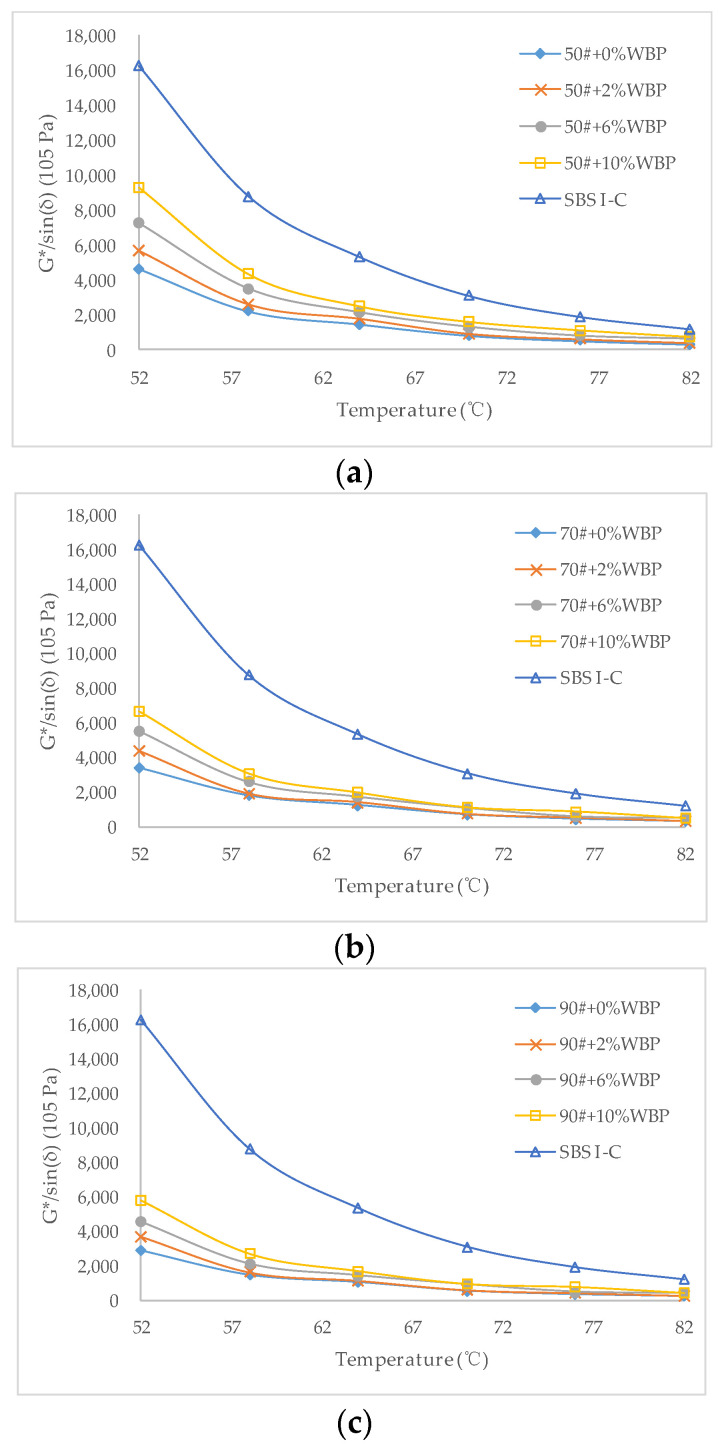
G*/sin(δ) of asphalt with different WBP content: (**a**) 50^#^ + 0%WBP, 50^#^ + 2%WBP, 50^#^ + 6%WBP, 50^#^ + 10%WBP, SBS Ι-C; (**b**) 70^#^ + 0%WBP, 70^#^ + 2%WBP, 70^#^ + 6%WBP, 70^#^ + 10%WBP, SBS Ι-C; (**c**) 90^#^ + 0%WBP, 90^#^ + 2%WBP, 90^#^ + 6%WBP, 90^#^ + 10%WBP, SBS Ι-C.

The G*/sin(δ) of different asphalt with different WBP content is shown in Figure 6.

The G*/sin(δ), as an index to evaluate the resistance of asphalt materials to permanent deformation, has been proven to be widely applicable. As shown in Figure 6, the G*/sin(δ) of various asphalts decreases rapidly with the increase in scanning temperature. For 50^#^ asphalt, the greater the content of WBP, the greater the G*/sin(δ) at the same scanning temperature. The G*/sin(δ) of asphalt with 10% WBP is about 1.5–2.0 times that of asphalt without WBP. The same rule exists for 70^#^ asphalt and 90^#^ asphalt, which is basically consistent with the change rule of dynamic shear modulus. The mechanism of the above phenomenon can still be attributed to the fact that the mixing of WBP has enhanced the aromatic C==C vibration, thereby enhancing the anti-deformation ability of asphalt under high-temperature conditions. Compared with 50^#^, 70^#^, and 90^#^ asphalt, when the content of WBP is the same, under the same scanning temperature, the G*/sin(δ) of 50^#^ asphalt is the largest, followed by 70^#^ asphalt, and 90^#^ asphalt the smallest. But by comparing SBS Ι-C modified asphalt, even if it is 50^#^ modified asphalt mixed with 10% WBP, it is still unable to put on a part with SBS Ι-C modified asphalt. The G*/sin(δ) of SBS Ι-C modified asphalt at the same scanning temperature is much higher than that of 50^#^ modified asphalt with 10% WBP. This is consistent with the rule of dynamic shear modulus in the first part of Section 3.2, which further suggests that WBP has a certain effect on improving the high-temperature performance of asphalt, but it cannot achieve the similar modification effect with SBS.

### 3.3. Full Section Fracture Energy

The full section fracture energy does not change with the loading rate, so it belongs to an attribute of the asphalt material itself. The FSFET is improved based on the direct tension (DT) test. This paper mainly tests the full section fracture energy of asphalt in the normal temperature region. The test temperature is 15 °C and the test rate is 500 mm/min. The full section fracture energy is equal to the envelope area from the initial stage of the actual stress-strain curve to the peak value corresponding to the actual stress-strain curve. The full section fracture energy can be used to reflect the low-temperature crack resistance of asphalt. It is generally believed that the higher the fracture energy density, the better the low-temperature crack resistance of asphalt, and the lower the fracture energy density, the worse the low-temperature crack resistance of asphalt.

**Figure 7:**
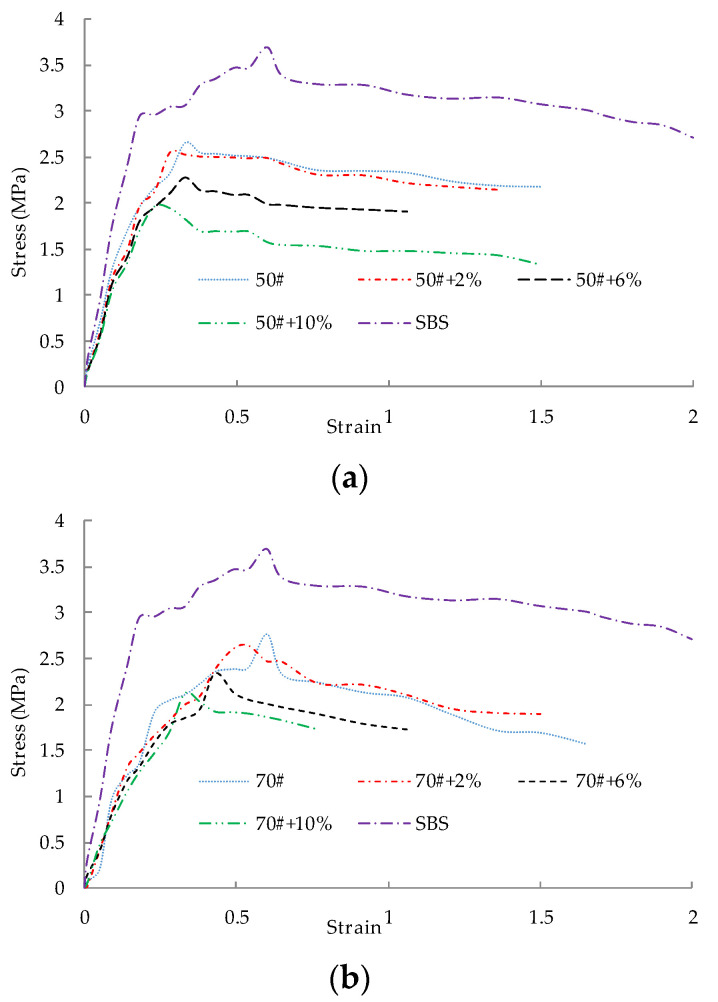

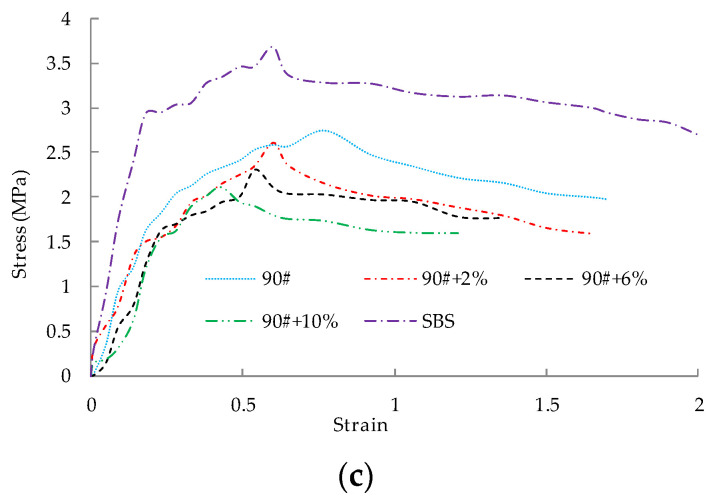
Stress-strain curves of 50^#^ asphalt with different spent battery powder content: (**a**) 50^#^ + 0%WBP, 50^#^ + 2%WBP, 50^#^ + 6%WBP, 50^#^ + 10%WBP, SBS Ι-C; (**b**) 70^#^ + 0%WBP, 70^#^ + 2%WBP, 70^#^ + 6%WBP, 70^#^ + 10%WBP, SBS Ι-C; (**c**) 90^#^ + 0%WBP, 90^#^ + 2%WBP, 90^#^ + 6%WBP, 90^#^ + 10%WBP, SBS Ι-C.

Figure 7 shows the actual stress-strain curve of different asphalts in the loading process with different WBP content.

Figure 7 shows that for the same grade asphalt when WBP is added, the peak value of the stress-strain curve of asphalt generally moves down. The greater the content of WBP is, the greater the peak value moves down; then the envelope area of the corresponding peak value of the stress-strain curve from the initial stage to the actual stress-strain curve also decreases. Therefore, the low-temperature crack resistance of asphalt decreases. For different asphalts with the same content of WBP, the larger the grade of the asphalt, the larger the envelope area from the initial stage of the stress-strain curve to the corresponding peak value of the actual stress-strain curve, indicating that its low-temperature crack resistance is also better. In addition, the corresponding envelope area of SBS Ι-C modified asphalt is larger than that of other types of asphalt, indicating that its low-temperature crack resistance is the best.

The full section fracture energy is equal to the envelope area from the initial stage of the actual stress-strain curve to the peak value corresponding to the actual stress-strain curve. When the first stress peak appears, the actual stress and actual strain can be calculated according to Equations (1) and (2) [22].

(1) $$\sigma =\frac{F}{A}=\frac{F}{A_{1}\cdot L_{1}/L}$$

(2) $$\varepsilon =\ln\frac{L}{L_{1}}$$

where: A_1_ is the area at the first stress peak of the central section; A is the area after the first stress peak; L is the length of the initial length before necking after the first stress peak; L_1_ is the length of the initial length before necking at the first stress peak; F is the stress.

Among the unknown parameters, the value of *A* can be calculated based on the principle of volume invariance before and after deformation, and the central section area (after the first stress peak) can be calculated by Equation (3) [22].

(3) $$A\cdot L=A_{1}\cdot L_{1}\Rightarrow A=A_{1}\cdot \frac{L_{1}}{L}$$

where A_1_ and L_1_ can be calculated by nonlinear finite element large deformation formula, L = L_1_ + necking elongation value. Table 5 shows the calculated values of asphalt fracture energy density based on stress-strain.

The rules described in Table 5 are consistent with Figure 7. The breaking energy density of SBS Ι-C modified asphalt is much higher than that of other types of asphalt. When the content of WBP is 2%, the fracture energy density of all kinds of asphalt is slightly lower than the original asphalt. When the content of WBP is 6%, the fracture energy density is about 60–80% of the original asphalt, but when the content of WBP reaches 10%, the fracture energy density has dropped to about 40–60% of the original asphalt. These phenomena suggest that, based on the consideration of low-temperature performance, the content of WBP should not be too large, and the specific upper limit of the amount should be determined according to the requirements of the specific project on the low-temperature performance of asphalt.

### 3.4. Pavement Performance of Asphalt Mixture

It has been pointed out previously that the mixing of WBP can significantly improve the high-temperature performance of asphalt and reduce its low-temperature performance to a certain extent. In order to further determine the pavement performances of WBP modified asphalt mixture, different asphalt with different WBP content were selected, and the rutting test, low temperature bending test, freeze-thaw split test, and immersion Marshall test were carried out under the same other materials and gradation. The dynamic stability (DS), maximum bending tensile strain (ε_B_), freeze-thaw splitting strength ratio (TSR), and residual stability (MS′) of asphalt mixture were obtained, and the influence of WBP on the pavement performance of asphalt mixture was compared and evaluated.

**Figure 8:**
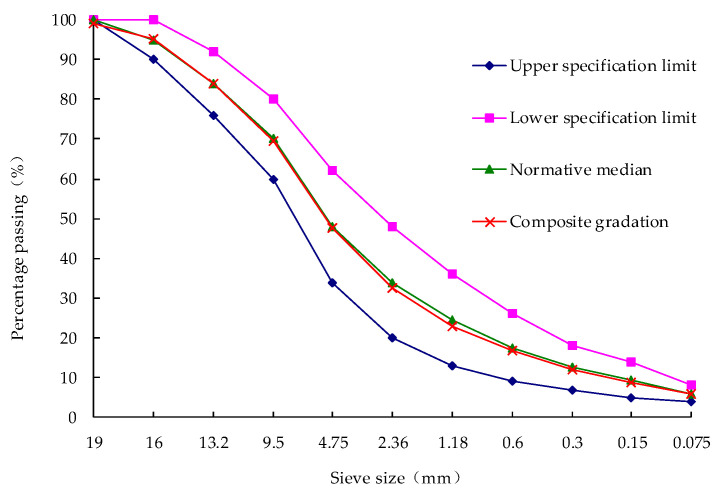
The curve of AC-16 gradation.

The grading type of asphalt mixture selected in this paper is dense graded bituminous mixture with a nominal maximum particle size 16 mm. And Figure 8 shows the curve of AC-16 gradation adopted in this paper.

Although the grading curves of all asphalt mixtures are the same, the asphalt aggregate ratio is different due to different asphalt types and WBP content. In this paper, according to the method of determining the optimum asphalt aggregate ratio, the asphalt aggregate ratio of various asphalt mixtures is determined. Table 6 is the optimum asphalt aggregate ratio of asphalt mixture

#### 3.4.1. High-Temperature Stability

The high-temperature stability of asphalt mixture mainly depends on the gradation of mineral mixture and the high-temperature bonding performance of asphalt. Because the gradation of the mineral mixture used in this paper is the same, the main factor affecting its high-temperature stability is the high-temperature bonding performance of asphalt.

**Figure 9:**
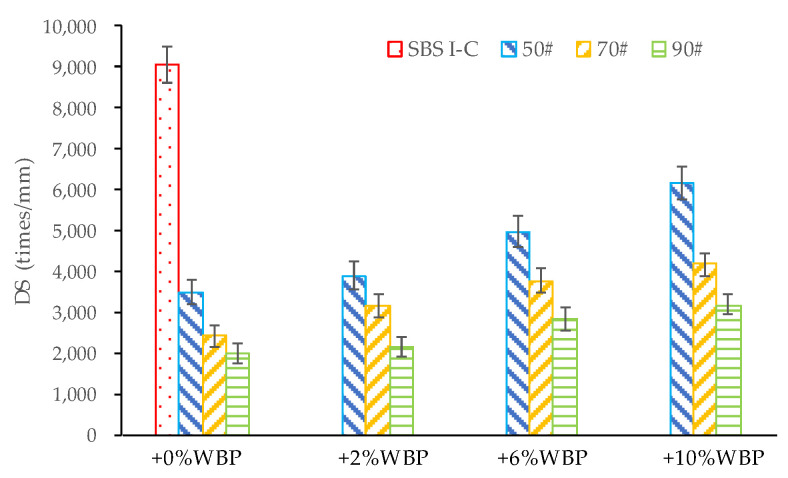
The DS of AC-16 asphalt mixtures, including different asphalt with different WBP content.

The temperature of the rutting test adopted in this paper is 60 °C, the wheel pressure is 0.7 MPa, the test time is 60 min, and the wheel pressure speed is 42 times ± 1 times/min. The DS test results of asphalt mixtures are shown in Figure 9.

Figure 9 shows that the DS of SBS-modified asphalt is much higher than that of WBP-modified asphalt mixture, indicating that there is a gap between WBP-modified asphalt mixture and SBS-modified asphalt mixture in terms of high-temperature stability. However, it is undeniable that the high-temperature performance of the asphalt mixture modified by WBP is also excellent. After adding WBP, the high-stability performance of the asphalt mixture has been greatly improved, and the DS of the asphalt mixture increases with the increase of the content of WBP. For asphalt with 6% WBP, the DS of asphalt mixture with 50^#^ + 6% WBP, 70^#^ + 6% WBP, and 90^#^ + 6% WBP have reached 4977 times/mm, 3758 times/mm, and 2845 times/mm, respectively, which can meet the requirements of various road surface courses on the high-temperature stability of asphalt mixture.

#### 3.4.2. Low-Temperature Anti-Cracking Performance

**Figure 10:**
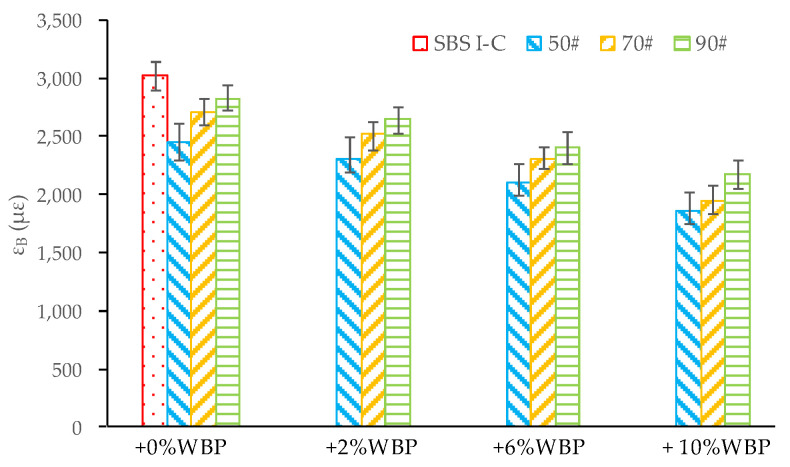
The ε_B_ of AC-16 asphalt mixtures, including different asphalt with different WBP content.

The low-temperature stiffness modulus of asphalt and the gradation of the asphalt mixture are the main factors affecting the low-temperature anti-cracking performance of the mixture. For this paper, the gradation of the asphalt mixture is the same, so the low-temperature stiffness modulus of asphalt becomes the main factor. In this paper, the low temperature bending test is used to evaluate the low temperature anti-cracking performance of asphalt mixture. The test temperature is −10 ± 0.5 °C and the loading rate is 50 mm/min. A concentrated load is applied to the midspan of the asphalt mixture beam specimen with the specified size until it is destroyed, and then the ε_B_ of the asphalt mixture when it is destroyed is calculated. The ε_B_ test results of asphalt mixtures are shown in Figure 10.

Figure 10 shows that the ε_B_ of asphalt mixture decreases gradually with the increasing content of WBP. For 50^#^ asphalt, compared with 0% WBP, the ε_B_ of 50^#^ + 2% WBP, 50^#^ + 6% WBP, 50^#^ + 10% WPB asphalt mixtures decreased by 6.5%, 16.5%, and 31.2%, respectively. For 70^#^ asphalt, compared with 0% WBP, the ε_B_ of 70^#^ + 2% WBP, 70^#^ + 6% WBP, 70^#^ + 10% WPB asphalt mixtures decreased by 7.3%, 16.9%, and 38.8% respectively. For 90^#^ asphalt, compared with 0% WBP, the ε_B_ of 90^#^ + 2% WBP, 90^#^ + 6% WBP, 90^#^ + 10% WPB asphalt mixture decreased by 6.5%, 17.4%, and 29.5%, respectively. Therefore, when selecting the content of WBP, it is necessary to consider its impact on the low-temperature anti-cracking performance of the mixture to meet the requirements of the specification for the low-temperature performance of the corresponding project area [35]. In addition, it is worth noting that the low-temperature anti-cracking performance of SBS modified asphalt mixture is still significantly superior to other asphalt mixtures.

#### 3.4.3. Water Stability

The adhesion of asphalt and aggregate is the main factor determining the water stability of the asphalt mixture. Since the aggregates selected in this paper are the same, the water stability of asphalt mixture of the same type of asphalt (such as 50^#^, 50^#^ + 2% WBP, 50^#^ + 6% WBP, and 50^#^ + 10% WBP) only depends on the adhesion of asphalt and aggregate. In this paper, the freeze-thaw splitting test and immersion Marshall test are used to obtain the TSR and MS′ of the asphalt mixture to evaluate its water stability.

**Figure 11:**
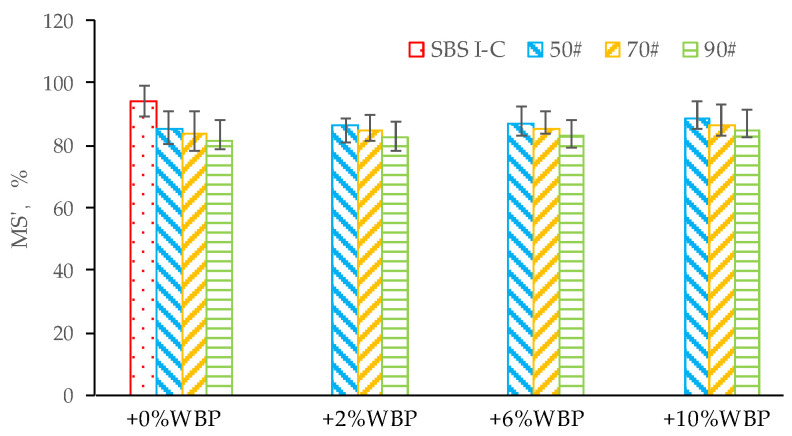
The MS’ of AC-16 asphalt mixtures, including different asphalt with different WBP content.

**Figure 12:**
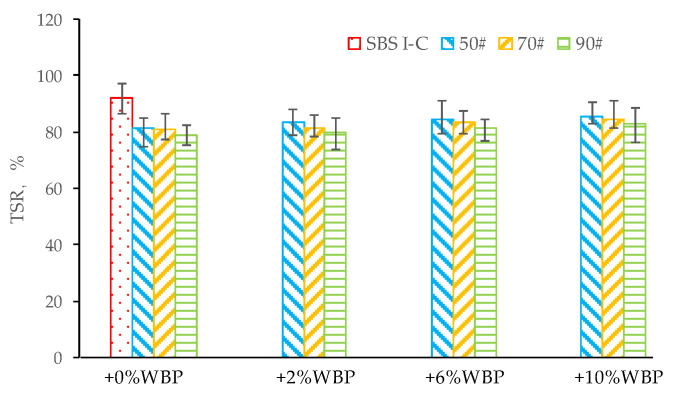
The TSR of AC-16 asphalt mixtures, including different asphalt with different WBP content.

Figure 11 and Figure 12 show the water stability test results of various asphalt mixtures.

Figure 11 and Figure 12 show that with the increasing content of WBP, the MS′, and TSR of asphalt mixture show a marginally increasing trend. For 50^#^ asphalt, compared with 0% WBP, the MS′ (TSR) of 50^#^ + 2% WBP, 50^#^ + 6% WBP, 50^#^ + 10% WPB asphalt mixtures increased by 1.5% (2.5%), 2.5% (3.9%), and 4.2% (5.0%), respectively; for 70^#^ asphalt, compared with 0% WBP, the MS′ (TSR) of 70^#^ + 2% WBP, 70^#^ + 6% WBP, 70^#^ + 10% WPB asphalt mixtures increased by 1.1% (0.9%), 2.2% (3.2%), and 3.6% (4.7%). For 90^#^ asphalt, compared with 0% WBP, the MS′ (TSR) of 90^#^ + 2% WBP, 90^#^ + 6% WBP, and 90^#^ + 10% WPB asphalt mixtures increased by 1.2% (1.5%), 2.0% (3.4%), and 4.0% (5.1%), respectively.

The test results show that with the increase of WBP content, the MS′, and TSR of the asphalt mixture increase slightly. This rule exists in different types of asphalt mixtures, such as 50^#^, 70^#^, and 90^#^ asphalt mixtures. Therefore, it is not difficult to draw a conclusion that the mixing of WBP can improve the water stability of the asphalt mixture to a certain extent, but the extent of improvement is limited. In addition, compared with SBS-modified asphalt mixture, the MS′ and TSR of 50^#^, 70^#^, and 90^#^ asphalt mixture with any WBP content are lower than those of SBS-modified asphalt mixture. So, it is not hard to get that the water stability of WBP modified asphalt mixture is relatively poor compared with SBS-modified asphalt mixture.

## 4. Conclusions

This paper studies the modification effect of WBP on asphalt. The FITR test of WBP, dynamic shear rheology test, and full section fracture energy test of asphalt are carried out. The high-temperature rheological properties and low-temperature properties of WBP modified asphalt are analyzed. The high-temperature stability, low-temperature crack resistance, and water stability of WBP-modified asphalt mixture are tested.

(1) There is no new characteristic absorption peak when WBP is mixed into asphalt, and the original characteristic peak has not disappeared. The modification of asphalt by WBP is essentially physical modification, but the mixing of WBP has a certain enhancement effect on the bond energy of the methylene group, which is helpful to improve the technical performance of modified asphalt.
(2) The greater the amount of WBP, the greater the dynamic shear modulus, and rutting factor under the same scanning temperature. The proportion of elastic components in asphalt can be significantly increased by adding WBP, thus enhancing the deformation resistance of asphalt under high-temperature conditions.
(3) The mixing of WBP reduces the proportion of viscous components in asphalt, which is unfavorable to the crack resistance under low temperatures.
(4) The greater the amount of WBP, the greater the downward shift of the peak value of the stress-strain curve of asphalt; the envelope area of the corresponding peak value from the initial stage of the stress-strain curve to the actual stress-strain curve also decreases, and the low-temperature cracking resistance of asphalt decreases.
(5) Compared with ordinary asphalt mixture, the high-temperature stability of WBP-modified asphalt mixture is significantly improved, and the water stability is slightly improved, but the low-temperature crack resistance is reduced to a certain extent.
(6) Although the WBP has significantly improved the high-temperature performance of asphalt, it is far from reaching the technical level of SBS Ι-C modified asphalt, and the high-temperature stability, low-temperature crack resistance, and water stability of the WBP-modified asphalt mixture cannot reach the level of SBS Ι-C modified asphalt mixture.

## Figures and Tables

**Table 1:** The technical indicators of various asphalt.

Test Items		50^#^	70^#^	90^#^	SBS Ι-C
Penetration at 25 °C (0.1 mm)		54.2	72.3	88.9	71.9
Softening point (°C)		52.4	48.7	46.8	64.6
Ductility at 5 °C (cm)			——	——	47.5
Ductility at 10 °C (cm)		17.2	28.5	45.3	——
RTFOT ^1^	Mass change (%)	0.2	−0.6	0.3	0.5
	Residual penetration ratio at 25 °C (%)	73.6	72.1	69.1	75.4
	Residual ductility at 5 °C (%)	9.4	16.9	20.2	——
	Residual ductility at 10 °C (%)	——	——	——	27.3

^1^ RTFOT: Rolling Thin Film Oven Test.

**Table 2:** The technical indicators of fine aggregate (particle size ≤ 2.36 mm).

Test Items					Test Result		
Apparent specific gravity					2.638		
Sand equivalent of fine aggregate (%)					60.0		
Angularity of fine aggregate (%)					52.2		
Sieve test results							
Sieve pore (mm)	4.75	2.36	1.18	0.6	0.3	0.15	0.075
Passing rate (%)	99.10	68.37	44.19	30.33	13.96	6.66	3.35

**Table 3:** The technical indicators of coarse aggregate (particle size ≥ 4.75 mm).

Test Items		Test Result
Crushing value (%)		12.5
Los Angeles Abrasion Loss (%)		19.4
Polished Stone Value (PSV)		43
Gross volume relative density	15~20 mm	2.633
	10~15 mm	2.606
	5~10 mm	2.594
Apparent specific gravity	15~20 mm	2.682
	10~15 mm	2.687
	5~10 mm	2.687
Water absorption (%)	15~20 mm	0.697
	10~15 mm	1.163
	5~10 mm	1.332
Content of acicular and flaky particles (%)	Mixture	10.6
	>9.5 mm	7.34
	<9.5 mm	11.84
Adhesion to asphalt (grade)		5

**Table 4:** The technical indicators of mineral powder.

Test Items	Test Result
apparent specific gravity	2.941
Particle size range < 0.6 mm (%) Particle size range < 0.15 mm (%) Particle size range < 0.075 mm (%)	99.98 99.82 99.17
Hydrophilicity coefficient	0.70

**Table 5:** Calculated value of fracture energy density in whole section of asphalt with different content of WBP.

Asphalt	0% + WBP	2% + WBP	6% + WBP	10% + WBP
50^#^	0.728	0.654	0.644	0.3325
70^#^	1.155	0.9996	0.803	0.699
90^#^	1.395	1.05	0.922	0.706
SBS	1.554	——	——	——

**Table 6:** The optimum asphalt aggregate ratio of AC-16 asphalt mixture.

Asphalt	0% + WBP	2% + WBP	6% + WBP	10% + WBP
50^#^	4.75	4.8	4.85	4.95
70^#^	4.75	4.75	4.85	4.9
90^#^	4.7	4.7	4.8	4.9
SBS	4.95	——	——	——

## Data Availability

Not applicable.
